# Constructing a critical thinking evaluation framework for college students majoring in the humanities

**DOI:** 10.3389/fpsyg.2022.1017885

**Published:** 2022-11-25

**Authors:** Suqi Li, Shenyu Tang, Xingyu Geng, Qi Liu

**Affiliations:** School of Education Science, Nanjing Normal University, Nanjing, China

**Keywords:** critical thinking, framework, college students, humanities, reliability and validity

## Abstract

**Introduction:**

Education for sustainable development (ESD) has focused on the promotion of sustainable thinking skills, capacities, or abilities for learners of different educational stages. Critical thinking (CT) plays an important role in the lifelong development of college students, which is also one of the key competencies in ESD. The development of a valuable framework for assessing college students’ CT is important for understanding their level of CT. Therefore, this study aimed to construct a reliable self-evaluation CT framework for college students majoring in the humanities.

**Methods:**

Exploratory factor analysis (EFA), confirmatory factor analysis (CFA), and Item analysis were conducted to explore the reliability and validity of the CT evaluation framework. Six hundred and forty-two college students majoring in the humanities were collected. The sample was randomly divided into two subsamples (*n*1 = 321, *n*2 = 321).

**Results:**

The Cronbach’s alpha coefficient for the whole scale was 0.909, and the values of the Cronbach’s alpha coefficients for individual factors of the scale ranged from 0.724 to 0.878. Then CFA was conducted within the scope of the validity study of the scale. In this way, the structure of the 7-factor scale was confirmed. Results indicated that the constructed evaluation framework performed consistently with the collected data. CFA also confirmed a good model fitting of the relevant 22 factors of the college students’ CT framework (*χ^2^/df* = 3.110, RMSEA = 0.056, GFI = 0.927, AGFI = 0.902, NFI = 0.923, and CFI = 0.946).

**Discussion:**

These findings revealed that the CT abilities self-evaluation scale was a valid and reliable instrument for measuring the CT abilities of college students in the humanities. Therefore, the college students’ CT self-evaluation framework included three dimensions: discipline cognition (DC), CT disposition, and CT skills. Among them, CT disposition consisted of motivation (MO), attention (AT), and open-mindedness (OM), while CT skills included clarification skills (CS), organization skills (OS), and reflection (RE). Therefore, this framework can be an effective instrument to support college students’ CT measurement. Consequently, some suggestions are also put forward regarding how to apply the instrument in future studies.

## Introduction

Nowadays, individuals should be equipped with the abilities of identifying problems, in-depth thinking, and generating effective solutions to cope with various risks and challenges caused by the rapid development of science and technology (Arisoy and Aybek, 2021). In this context, critical thinking (CT) is gaining increasing attention. Promoting college students’ CT is an important way of improving their abilities of problem solving and decision making to further enhance their lifelong development (Feng et al., 2010). Although human beings are not born with CT abilities (Scriven and Paul, 2005), they can be acquired through learning and training, and are always sustainable (Barta et al., 2022).

Especially in the field of education, CT should be valued (Pnevmatikos et al., 2019). Students should be good thinkers who possess the abilities of applying critical evaluation, finding, and collating evidence for their views, as well as maintaining a doubting attitude regarding the validity of facts provided by their teachers or other students (Sulaiman et al., 2010). Many countries have regarded the development of students’ CT as one of the fundamental educational goals (Flores et al., 2012; Ennis, 2018). CT is helpful for students to develop their constructive, creative, and productive thinking, as well as to foster their independence (Wechsler et al., 2018; Odebiyi and Odebiyi, 2021). It also provides the power to broaden their horizons (Les and Moroz, 2021). Meanwhile, when college students have a high level of CT abilities, they will likely perform better in their future careers (Stone et al., 2017; Cáceres et al., 2020). Therefore, college students should be capable of learning to access knowledge, solve problems, and embrace different ideas to develop their CT ability (Ulger, 2018; Arisoy and Aybek, 2021).

Due to the significant meaningfulness of CT abilities at all education levels and in various disciplines, how to cultivate students’ CT abilities has been the focus of CT-related research (Fernández-Santín and Feliu-Torruella, 2020). Many studies have shown that inquiry-based learning activities or programs are an effective way to exercise and enhance students’ CT abilities (Thaiposri and Wannapiroon, 2015; Liang and Fung, 2020; Boso et al., 2021; Chen et al., 2022). Students not only need the motivation and belief to actively participate in such learning activities and to commit to problem solving, but also need the learning skills to cope with the problems that may be encountered in problem-solving oriented learning activities. These requirements are in line with the cultivation of students’ CT abilities. Meanwhile, research has also indicated that there is an interrelationship between problem solving and CT (Dunne, 2015; Kanbay and Okanlı, 2017).

However, another important issue is how to test whether learning activities contribute to improving the level of students’ CT abilities. It is effective to measure students’ CT abilities through using CT measurement instruments. Some CT measurement frameworks have been developed to cope with the need to cultivate CT abilities in teaching and learning activities (Saad and Zainudin, 2022). However, there are still some imperfections in these existing CT evaluation frameworks. For example, most studies on college students’ CT are in the field of science, with very little research on students in the humanities, and even less on specifically developing CT assessment frameworks for college students in the humanities. Only Khandaghi et al. (2011) conducted a study on the CT disposition of college students in the humanities, and the result indicated that their CT abilities were at an intermediate level. However, there are few descriptions of college students’ CT with a background in humanities disciplines. Compared to humanities disciplines, science disciplines seem to place more emphasis on logical and rational thinking, which might cater more to the development of CT abilities (Li, 2021). However, it is also vital for college students in the humanities to engage in rational thinking processes (Al-Khatib, 2019). Hence, it is worth performing CT abilities evaluations of college students in the humanities by constructing a CT evaluation framework specifically for such students. In addition, previous measurements of CT have tended to be constructed according to one dimension of CT only, either CT skills or CT disposition. CT skills and disposition are equally important factors, and the level of CT abilities can be assessed more comprehensively and accurately by measuring both dimensions simultaneously. Therefore, the purpose of this study was to develop a self-evaluation CT framework for college students that integrates both CT skills and disposition dimensions to comprehensively evaluate the CT ability of college students in the humanities.

## Literature review

### CT of college students in the humanities

CT is hardly a new concept, as it can be traced back 2,500 years to the dialogs of Socrates (Giannouli and Giannoulis, 2021). In the book, *How We Think,* Dewey (1933, p 9; first edition, 1910) mentioned that thinking critically can help us move forward in our thinking. Subsequently, different explanations of CT have been presented through different perspectives by researchers. Some researchers think that CT means to think with logic and reasonableness (Mulnix and Mulnix, 2010), while others suggest that CT refers to the specific learning process in which learners need to think critically to achieve learning objectives through making decisions and problem solving (Ennis, 1987).

Generally, for a consensus, CT involves two aspects: CT skills and CT disposition (Bensley et al., 2010; Sosu, 2013). CT skills refer to the abilities to understand problems and produce reasonable solutions to problems, such as analysis, interpretation, and the drawing of conclusions (Chan, 2019; Ahmady and Shahbazi, 2020). CT disposition emphasizes the willingness of individuals to apply the skills mentioned above when there is a problem or issue that needs to be solved (Chen et al., 2020). People are urged by CT disposition to engage in a reflective, inferential thinking process about the information they receive (Álvarez-Huerta et al., 2022), and then in specific problem-solving processes, specific CT skills would be applied. CT disposition is the motivation for critical behavior and an important quality for the learning and use of critical skills (Lederer, 2007; Jiang et al., 2018).

For college students, the cultivation of their CT abilities is usually based on specific learning curriculums (O’Reilly et al., 2022). Hence, many studies about students’ CT have been conducted in various disciplines. For example, in science education, Ma et al.’s (2021) study confirmed that there was a significant relationship between CT and science achievement, so they suggested that it might be valuable to consider fostering CT as a considerable outcome in science education. In political science, when developing college students’ CT, teachers should focus on not only the development of skills, but also of meta-awareness (Berdahl et al., 2021), which emphasizes the importance of CT disposition, i.e., learners not only need to acquire CT skills, such as analysis, inference, and interpretation, but also need to have clear cognition of how to apply these skills at a cognitive level. Duro et al. (2013) found that psychology students valued explicit CT training. For students majoring in mathematics, Basri and Rahman (2019) developed an assessment framework to investigate students’ CT when solving mathematical problems. According to the above literature review, there have been many studies on CT in various disciplines, which also reflects the significant importance of CT for the development of students in various disciplines. However, most studies on CT have been conducted in the field of science subjects, such as mathematics, business, nursing, and so on (Kim et al., 2014; Siew and Mapeala, 2016; Basri and Rahman, 2019), but there have been few studies on the CT of students in the humanities (Ennis, 2018).

There is a widespread stereotype that compared to humanities subjects, science majors are more logical, and so more attention should be paid to their CT (Lin, 2016). This begs the question, are all students in the humanities (e.g., history, pedagogy, Chinese language literature, and so on) sensual or “romantic”? Do they not also need to develop independent, logical, and CT? Can they depend only on “romantic” thinking? This may be a prejudice. In fact, the humanities are subjects that focus on humanities and our society (Lin, 2020). Humanities should be seen as the purpose rather than as a tool. The academic literacy of humanities needs to be developed and enhanced through a long-term, subtle learning process (Bhatt and Samanhudi, 2022), and the significance for individuals is profound. Hence, the subjects of both humanities and sciences play an equally important role in an individual’s lifelong development. As such, what should students majoring in humanities subjects do to develop and enhance their professional competence? Chen and Wei (2021) suggested that individuals in the humanities should have the abilities to identify and tackle unstructured problems to adapt to the changing environments, and this suggestion is in line with a developmental pathway for fostering CT. Therefore, developing their CT abilities is an important way to foster the humanistic literacy of students in the humanities. Specifically, it is important to be equipped with the abilities to think independently and questioningly, to read individually, and to interpret texts in depth and in multiple senses. They also need to learn and understand the content of texts and evaluate the views of others in order to expand the breadth of their thinking (Barrett, 2005). Moreover, they need the ability to analyze issues dialectically and rationally, and to continually reflect on themselves and offer constructive comments (Klugman, 2018; Dumitru, 2019). Collegiate CT skills are taught *via* independent courses or embedded modules (Zhang et al., 2022). The humanities are no exception. Yang (2007) once designed thematic history projects, as independent courses, to foster students’ disposition toward CT concerning the subject of history, and the results showed that the history projects can support learners’ development of historical literacy and CT. In a word, the humanities also play an important role in fostering the development and enhancement of college students’ CT, esthetic appreciation and creativity, and cultural heritage and understanding (Jomli et al., 2021). Having good CT therefore also plays a crucial role in the lifelong development of students in the humanities.

An accurate assessment of the level of CT abilities is an important prerequisite for targeted improvement of students’ CT abilities in special disciplines (Braeuning et al., 2021). Therefore, it might be meaningful to construct a self-evaluation CT framework for college students in the humanities according to their professional traits.

### Evaluating college students’ CT

Given that CT can be cultivated (Butler et al., 2017), more attention has been paid to how to improve students’ CT abilities level in instruction and learning (Araya, 2020; Suh et al., 2021). However, it is also important to examine how CT can be better assessed. The evaluation of thinking is helpful for students to think at higher levels (Kilic et al., 2020). Although the definitions of CT are controversial (Hashemi and Ghanizadeh, 2012), many researchers have reached a consensus on the main components of CT: skills and disposition (Bensley et al., 2016), and different CT evaluation frameworks have been developed according to one of the two dimensions. For example, Li and Liu (2021) developed a five-skill framework for high school students which included analysis, inference, evaluation, construct, and self-reflection. Meanwhile, in recent years, the assessment of CT disposition has also attracted the interest of a growing number of researchers. Sosu (2013) developed the “Critical Thinking Disposition Scale” (STDS), which included two dimensions: critical openness and reflective skepticism. The specific taxonomies of the evaluation framework of CT skills and dispositions is shown in Table 1. As illustrated in Table 1, there are some universal core items to describe CT skills. For the dimension of CT skills, the sub-dimensions of interpretation, analysis, inference, and evaluation are the important components. Those CT skills are usually applied along with the general process of learning activities (Hsu et al., 2022). For instance, at the beginning of learning activities, students should have a clear understanding of the issues raised and the knowledge utilized through applying interpretation skills. Likewise, there are some universal core items to describe CT dispositions, such as open-mindedness, attentiveness, flexibility, curiosity, and so on.

**Table 1:** Taxonomies of the evaluation framework of CT skills and dispositions.

Core dimensions	Sources	Core items
Critical thinking skills	Thomas and Lok (2015)	Interpretation Explanation Analysis Inference Evaluation Self-regulation
	Dwyer et al. (2014), pp. 43–52	Analysis Evaluation Inference Reflective Judgement
	Simpson and Courtney (2002), pp. 9–10	Interpretation Analysis Inference Explanation Evaluation
	Abrami et al. (2008)	Interpreting Predicting Analyzing Evaluating
	Murphy (2004)	Recognize Understand Analyze Evaluate Create
	Bellaera et al. (2021)	Analyze Creativity Deductive reasoning Description Evaluation Explanation Inductive reasoning Inference Interpretation Problem-solving
	Li and Liu (2021)	analysis inference evaluation construct self-reflection
Critical thinking dispositions	Facione et al. (1994)	Curiosity inquisitiveness open-mindedness decision-making
	Facione (1990)	Inquisitiveness Well-informed Open-mindedness Precision Flexibility Persistence
	Perkins et al. (1993)	Adventure Wondering Explanation Strategy Metacognitive
	Ennis (1962)	Well-informed Open-mindedness Determination Overall situation thought
	Halpern (1998)	Open-mindedness Awareness Persistence Self-correct Programmatic
	Quinn et al. (2020)	Intrinsic Attentiveness Open-Mindedness Perseverance Reflection Organization

For a good critical thinker, it is equally important to have both dispositional CT and CT skills. Students need to have the awareness of applying CT abilities to think about problem-solving and subsequently be able to utilize a variety of CT skills in specific problem-solving processes. Therefore, we argue that designing a CT self-evaluation framework that integrates the two dimensions will provide a more comprehensive assessment of college students’ CT. In terms of CT disposition, motivation, attentiveness, and open-mindedness were included as the three sub-dimensions of CT disposition. Motivation is an important prerequisite for all thinking activities (Rodríguez-Sabiote et al., 2022). Especially in problem-solving-oriented learning activities, the development of CT abilities will be significantly influenced by the motivation level (Berestova et al., 2021). Attentiveness refers to the state of concentration of the learner during the learning process, which reflects the learners’ level of commitment to learning, playing a crucial role in the development of CT abilities during the learning process. Open-mindedness requires learners to keep an open mind to the views of others when engaging in learning activities. The three sub-dimensions have been used to reflect leaners’ disposition to think critically. Especially in the humanities, it is only through in-depth communication between learners that a crash of minds and an improvement in abilities can take place (Liu et al., 2022), and it is therefore essential that learners maintain a high level of motivation, attentiveness, and open-mindedness in this process to develop their CT abilities. In terms of CT skills, three sub-dimensions were also selected to measure the level of learners’ CT skills, namely clarification skills, organization skills, and reflection. In the humanities, it should be essential abilities for students to understand, analyze, and describe the literature and problems comprehensively and exactly (Chen and Wei, 2021). Then, following the ability to extract key information about the problem, to organize and process it, and to organize the information with the help of organizational tools such as diagrams and mind maps. Finally, the whole process of problem solving is reflected upon and evaluated (Ghanizadeh, 2016), and research has shown that reflection learning intervention could significantly improve learners’ CT abilities (Chen et al., 2019).

### Research purpose

CT plays an important role in college students’ academic and lifelong career development (Din, 2020). In the current study on college students’ CT measurement, it can be improved in two main ways.

Firstly, the attention to the discipline cognition related to CT in previous studies is insufficient. Generally, students’ CT abilities can be cultivated based on two contexts: the subject-specific instructional context and the general skills instructional context (Ennis, 1989; Swartz, 2018). In authentic teaching and learning contexts, the generation and development of CT usually takes place in problem-oriented learning activities (Liang and Fung, 2020), in which students need to achieve their learning objectives by identifying problems and solving them. According to Willingham (2007), if you are to think critically, you must have a sound knowledge base of the problem or topic of enquiry and view it from multiple perspectives. Due to the difference in nature of the disciplines, the format of specific learning activities should also vary. Hence, an adequate cognition of the discipline is an important prerequisite for learning activities; meanwhile, college students’ cognition level regarding their discipline should also be an important assessment criterion for them to understand their own level of CT abilities. Cognition refers to the acquisition of knowledge through mental activity (e.g., forming concepts, perceptions, judgments, or imagination; Colling et al., 2022). Learners’ thinking, beliefs, and feelings will affect how they behave (Han et al., 2021). Analogically speaking, discipline cognition refers to an individual’s understanding of their discipline’s backgrounds and knowledge (Flynn et al., 2021). Cognition should be an important variable in CT instruction (Ma and Luo, 2020). In the current study, we added the dimension of discipline cognition into the self-evaluation CT framework of college students in the humanities. What’s more, in order to represent the learning contexts of humanities disciplines, the specific descriptions of items are concerned with the knowledge of the humanities, (e.g., “I can recognize the strengths and limitations of the discipline I am majoring in.,” and “Through studying this subject, my understanding of the world and life is constantly developing.”).

Secondly, the measurement factors of CT skills and disposition should be more specific according to the specific humanities background. In previous studies, researchers tended to measure students’ CT in terms of one of the two dimensions of CT skills. CT thinking skills used to be measured from perspectives such as analysis, interpretation, inference, self-regulation, and evaluation. However, in specific learning processes, how should students concretely analyze and interpret the problems they encounter, and how can they self-regulate their learning processes and evaluate their learning outcomes? Those issues should also be considered to evaluate college students’ levels of CT abilities more accurately. Therefore, the current study attempted to construct a CT framework in a more specific way, and by integrating both dimensions of CT disposition and skills. Therefore, what specific factors would work well as dimensions for evaluating the CT abilities of college students in the humanities? In the current study, firstly, students’ disposition to think critically is assessed in terms of three sub-dimensions: motivation, attention, and open-mindedness, to help students understand the strength of their own awareness to engage in CT (Bravo et al., 2020). Motivation is an important prerequisite for all thinking activities (Rodríguez-Sabiote et al., 2022), and it could contribute to the development of engagement, behavior, and analysis of problems (Berestova et al., 2021). Meanwhile, there was a positive relationship between academic motivation and CT. Therefore, in the current study, motivation is still one of the crucial factors. The sub-dimension of attentiveness was also an important measurement factor, which aimed to investigate the level of the persistence of attention. Attentiveness also has a positive influence on a variety of student behaviors (Reynolds, 2008), while the sub-dimension of open-mindedness mainly assesses college students’ flexibility of thinking, which is also an important factor of CT (Southworth, 2020). A good critical thinker should be receptive of some views that might be challenging to their own prior beliefs with an open-minded attitude (Southworth, 2022). Secondly, college students’ CT skills were then assessed in the following three sub-dimensions of clarification skills, organization skills, and reflection, with the aim of understanding how well students use CT skills in the problem-solving process (Tumkaya et al., 2009). The three sub-dimensions of CT skills selected in this framework are consistent with the specific learning process of problem solving, which begins with a clear description and understanding of the problem, i.e., clarification skills. In the humanities, it should be an essential competence for students to understand, analyze, and describe the literature and problems comprehensively and exactly (Chen and Wei, 2021).

**Figure 1:**
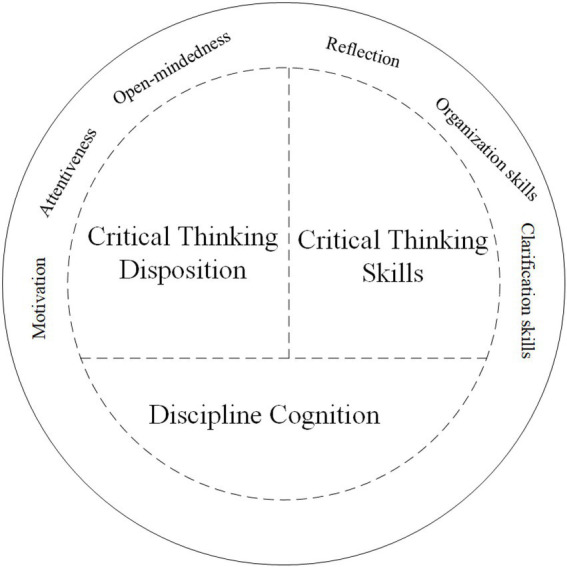
A model for evaluating the CT abilities of college students in the humanities.

We thus constructed a model for evaluating the CT of college students in the humanities (see Figure 1). The proposed evaluation framework incorporates three dimensions: discipline cognition (DC), CT disposition, and CT skills. Among them, CT disposition includes the three sub-dimensions of motivation (MO), attention (AT), and open-mindedness (OM), while CT skills include the three sub-dimensions of clarification skills (CS), organization skills (OS), and reflection (RE). In other words, this study aimed to construct a seven-dimensional evaluation framework and to test whether it is an effective instrument for measuring the CT of college students in the humanities.

## Materials and methods

### Research design

In order to address the two problems of the existing college students’ CT evaluation frameworks mentioned above, a CT self-evaluation framework for college students in the humanities was preliminarily developed in this study, including the following seven factors: discipline cognition (2 items), motivation (5 items), attentiveness (5 items), open-mindedness (5 items), clarification skills (3 items), organization (3 items), and reflection (4 items).

Then, to ensure the content validity of the measurement framework, four experts who have studied CT and five teachers who have worked in the field of humanities were invited to review all items and give feedback. The research team compared the similarities and differences in expert opinions and made joint decisions. Meanwhile, to ensure the popularity, accuracy, and objectivity of the items, 25 college students majoring in humanities participated in the pretest, and the presentation and description of the items was improved according to their feedback. Finally, a questionnaire consisting of 30 items was constructed, including three items for participants’ socio-demographic information (e.g., gender, grade, and subject), two for discipline cognition, five for motivation, five for attention, five for open-mindedness, three for clarification skills, three for organization skills, and four for reflection (as shown in Table 2). For each item, a 5-point Likert-style scale (5 = strongly agree, 4 = agree, 3 = neutral, 2 = disagree, 1 = strongly disagree) was used.

**Table 2:** Dimensions and items of the college students’ CTS evaluation framework.

Dimension	No.	Item	Code	Source
Discipline Cognition (DC)	1	I can recognize the strengths and limitations of the discipline I am majoring in.	DC1	Self-compiled based on the disciplinary characteristics of the humanities
	2	Through studying this subject, my understanding of the world and life is constantly developing	DC2	
Motivation (MO)	1	I look forward to learning challenging things	MO1	Nair and Stamler (2013)
	2	Completing difficult tasks is fun for me	MO2	
	3	Even if material is difficult to comprehend, I enjoy dealing with information that arouses my curiosity	MO3	
	4	I enjoy figuring out how things work	MO4	
	5	Being inquisitive is one of my strong points	MO5	
Attentiveness (AT)	1	I find that I’m easily distracted when thinking about a task	AT1	Nair and Stamler (2013)
	2	I find it hard to concentrate when thinking about problems	AT2	
	3	I often miss out on important information because I’m thinking of other things	AT3	
	4	I often daydream when learning a new topic	AT4	
	5	I get so bored with things that I quit before I finish what I planned to do	AT5	
Open-mindedness (OM)	1	Thinking is not about ‘being flexible’, it’s about being right	OM1	Bentler (1990)
	2	Being open-minded about different world views is less important than people think	OM2	
	3	When attempting to solve complex problems, it’s better to give up fast, if you cannot reach a solution so quickly	OM3	
	4	Breaking a problem down into smaller parts makes the problem more difficult	OM4	
	5	I know what I think and believe, so it’s important not to dwell on it any further	OM5	
Clarification skills (CL)	1	When I’m thinking, my mind always remains clear	CL1	Quinn et al. (2020)
	2	When a subject is talked about, I think that it’s important to define abstract concepts	CL2	
	3	Making a complex idea clear and easy to understand is very important	CL3	
Organization skills (OR)	1	I like to make a list of things I need to do and thoughts I may have	OS1	Quinn et al. (2020)
	2	When I’m reading or in class, I like to take notes to organize my thoughts in a timely manner	OS2	
	3	I like to make various charts to organize a great deal of knowledge I’ve learnt and ideas I’ve generated	OS3	
Reflection (RE)	1	When a theory, interpretation, or conclusion is presented to me, I try to decide if there is good supporting evidence	RE1	Kember et al. (2010)
	2	When faced with a decision, I seek as much information as possible	RE2	
	3	I try to gather as much information about a topic before I draw a conclusion about it	RE3	
	4	Putting together the information I have gathered from various sources helps me to make better choices	RE4	

### Participants and data collection

In the current study, simple random sampling was adopted and the online questionnaire was uploaded on Questionnaire Star^1^ (accessed on 18 March 2022), a professional online survey tool widely used in China (Sarjinder, 2003). The link to the online questionnaire was sent to the teachers in the humanities of some colleges in Jiangsu, China. Then teachers sent the link to their students. In the first part of the questionnaire, students were told that they were participating in an anonymous study, the content of which may be published without any commercial use. If they did not want to participate in the survey, they could quit the website of the online questionnaire. Students who agreed to participate in the survey filled in the questionnaire. In addition, to ensure the reliability of the results of the subsequent data analysis, the ratio of the number of questionnaire items to the number of participants should be 1:5, and the larger the sample size the better (Gorsuch, 1983). Therefore, eventually, 654 college students agreed to take part in the study, and completed the online questionnaire. After deleting those questionnaires with the same answer for all items or overly short response times, the effective number of samples was 642, with an effective rate of 98.2%.

The recruited effective sample comprised 642 participants, of whom 67.4% were female (*n* = 433), and 32.6% were male (*n* = 209). Sophomores (*n* = 215, 33.5%) and juniors (*n* = 249, 38.8%) made up most of the total number of participants. Meanwhile, the current study aimed to construct a CT framework for college students in the humanities field; hence, all participants were students in humanities disciplines, such as history (*n* = 187, 29.1%), educational history (*n* = 78, 12.2%), philosophy (*n* = 97, 15.1%), Chinese language and literature (*n* = 221, 34.4%), and pedagogy (*n* = 59, 9.2%). The specific socio-demographic information is shown in Table 3.

**Table 3:** Socio-demographic profile of respondents.

Sociodemographic	Characteristics	*N*	%
Gender	Male	209	32.6
	Female	433	67.4
Grade	Freshman	91	14.2
	Sophomore	215	33.5
	Junior	249	38.8
	Senior	87	13.5
Subject	History	187	29.1
	Education history	78	12.2
	Philosophy	97	15.1
	Chinese language and literature	221	34.4
	Pedagogy	59	9.2

### Data analysis

To construct an evaluation framework of college students’ CT skills and to confirm its reliability and validity, exploratory factor analysis (EFA), confirmatory factor analysis (CFA), and item analysis were carried out. Firstly, 642 samples were randomly assigned to two groups, with 321 samples in each (Yurdakul et al., 2012) to avoid inflation of the Cronbach’s alpha value or other effects (Devellis, 2011). EFA was used to analyze the first group of samples. CFA was applied to the second sample. Firstly, EFA was conducted in order to determine the underlying factor structure of the CT-evaluation framework and to make decisions about item retention (Kieffer, 1988). During this process, principal component analysis (PCA) was applied as an EFA factor extraction technique (Vogel et al., 2009). CFA was then used to confirm the factor structure of the scale using the second group of 321 samples (Kline, 2005). Lastly, all samples were analyzed to test the differentiation and suitability of the items (Yurdakul et al., 2012). SPSS 18.0 and AMOS 24.0 were applied to analyze the collected data.

## Results

### EFA

SPSS 22.0 was used for conducting EFA, and the maximum variance method was adopted for factor rotation.

#### Reliability analysis of the scale

Prior to the EFA, sample variance and sample size evaluations were conducted. An evaluation of Bartlett’s Test of Sphericity was found to be significant, thus confirming homogeneity of variance (*χ^2^* = 9162.198; *p* < 0.001). Then, the Cronbach’s alpha value (Pallant, 2007) was applied to evaluate the reliability of the scale, and the results showed that the whole scale had good reliability (*α* = 0.909). Specifically, the Cronbach’s alpha values of the seven factors were 0.724 (DC), 0.771 (MO), 0.878 (AT), 0.839 (OM), 0.819 (CL), 0.755 (OR), and 0.878 (RE), indicating their reliability. The Kaiser-Meyer Olkin (KMO) value of the questionnaire was 0.907, showing the appropriateness of the EFA (Kaiser, 1974).

#### Validity analysis of the scale

To confirm the validity of the evaluation dimensions, the method of PCA was applied to extract factors, and maximum variance rotation was used for the EFA. Seven factors were finally obtained. Kieffer (1988) suggested that two strategies should be applied for EFA. Thus, oblique rotation and orthogonal rotation were both used. If the results of the two methods are similar, the results obtained by the orthogonal rotation method can be used. Therefore, in the current study, two methods were both applied for EFA, namely optimal skew and maximum variance orthogonal rotation. The results of the two methods showed no significant difference. This study thus applied the results of the maximum variance orthogonal rotation method. MO5, OM4, and OM5 were removed since their maximum factor loadings were not in line with their initial evaluation dimension (Conway and Huffcutt, 2016). In addition, the factors with an eigenvalue higher than 1 were picked. Items with a factor loading of less than 0.4 and with inconsistent content were removed through the multiple orthogonal rotations (Zhao et al., 2021). There were 25 items with eigenvalues greater than 1 and independent factor loadings greater than 0.5 which were retained (Fabrigar et al., 1999). Table 4 presents the results of the component transformation matrix. Finally, seven factors were selected, with a cumulative variance contribution of 71.413% (Conway and Huffcutt, 2016). The eigenvalues and cumulative variance contributions of the seven factors are shown in Table 5.

**Table 4:** The factor analysis of college students’ CT framework (*N* = 321).

Items	1	2	3	4	5	6	7
RE1	0.725						
RE2	0.859						
RE3	0.829						
RE4	0.807						
RE5	0.665						
AT1		0.836					
AT 2		0.846					
AT 3		0.801					
AT 4		0.819					
AT 5		0.784					
MO1			0.500				
MO2			0.740				
MO3			0.781				
MO4			0.798				
CL1				0.822			
CL2				0.773			
CL3				0.724			
OM 1					0.783		
OM 2					0.826		
OM 3					0.657		
OR1						0.797	
OR2						0.768	
OR3						0.780	
DC1							0.774
DC2							0.768

**Table 5:** The eigenvalues and contribution rates of the five factors in the model.

Component	Eigenvalue	Percentage of variance	Cumulative variance contribution rate
1	3.574	14.295%	14.295%
2	3.401	13.602%	27.897%
3	2.617	10.466%	38.364%
4	2.334	9.336%	47.700%
5	2.177	8.707%	56.406%
6	2.172	8.688%	65.094%
7	1.580	6.319%	71.413%

### CFA

The first-order CFA was adopted to determine the validity, convergence, and identifiability of the framework in this study (Kline, 2005). CFA was used to explore the relationships between each factor, and then to construct the evaluation framework of humanities college students’ CT.

#### Fitting validity analysis for the framework

**Figure 2:**
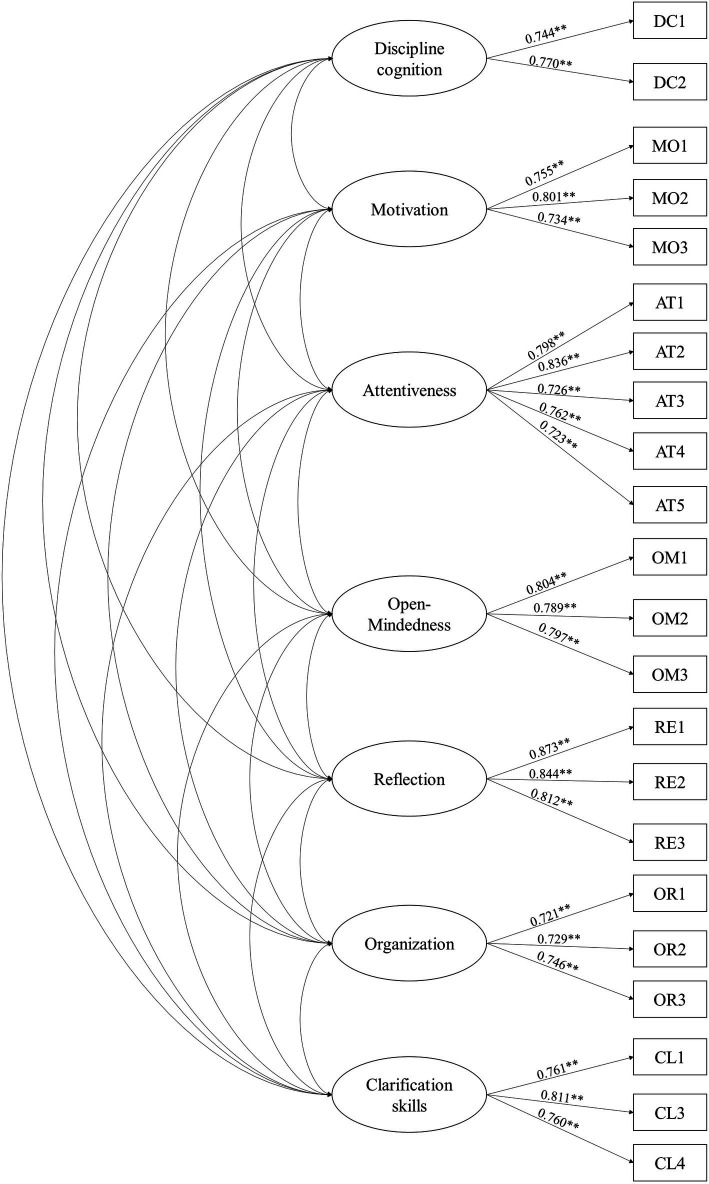
The first-order CFA model.

As shown in Figure 2, first-order CFA was conducted. According to Hair et al. (2014), items that do not meet the standard load (<0.5) must be eliminated. The absolute and relative fitting indexes were applied to verify the framework fit. The Chi-square/*df* in this research was 3.651, and the value of RMSEA was 0.044 (<0.08; Liu et al., 2021). In addition, the goodness-of-fit index (GFI) and adjusted fitness index (AGFI) were 0.923 and 0.906 respectively, which both met the reference standard proposed by Foster et al. (1993). Moreover, consistent with Hair et al. (2014) recommendations, the normed fitness index (NFI), comparative fitness index (CFI), incremental fitness index (IFI), and relative fitness index (RFI) were 0.975, 0.982, and 0.972 (>0.9). In addition, the values of simplifying the specification fitness index (PNFI), and streamlining fitness indicator (PGFI) were more than 0.5. Therefore, these results indicated the good fitting validity of the framework (Table 6).

**Table 6:** The fitting index of the evaluation framework.

Type	Fitting index	Threshold	Values	Results
Absolute fit index	Chi-square/*df*	<5	3.110	Acceptable
	RMSEA	<0.08	0.056	Acceptable
	GFI	>0.8	0.927	Acceptable
	AGFI	>0.8	0.902	Acceptable
Comparative fit index Incremental fit index	NFI	>0.9	0.923	Acceptable
	CFI	>0.9	0.946	Acceptable
	IFI	>0.9	0.947	Acceptable
	TLI	>0.9	0.934	Acceptable
Streaming fit index	PNFI	>0.5	0.751	Acceptable
Parsimonious fit index	PGFI	>0.5	0.689	Acceptable

#### Convergence validity analysis for the framework

The CFA results are shown in Table 7. The comprehensive reliability (CR) and average variance extracted (AVE) were used to test the construct validity of the framework. According to Hair et al. (2014), the CR value of all items should be more than 0.7. Thus, the CR of the 22 remaining items was good. What is more, Fornell and Larcker (1981) pointed out that if the AVE is higher than 0.5, the framework shows good convergence validity. Therefore, the results in Table 5 show that this evaluation framework has high validity and is reasonable.

**Table 7:** Results of the confirmatory factor analysis.

Potential variable	Item	Normalized factor loading	CR	AVE
Discipline Cognition (DC)	DC1	0.744	0.7287	0.5732
	DC2	0.770		
Organization skills (OR)	OR1	0.721	0.776	0.5359
	OR2	0.729		
	OR3	0.746		
Clarification skills (CL)	CL1	0.761	0.821	0.6048
	CL3	0.811		
	CL4	0.760		
Reflection (RE)	RE 1	0.873	0.8807	0.7113
	RE 2	0.844		
	RE 3	0.812		
Motivation (MO)	MO 1	0.755	0.8076	0.5835
	MO 2	0.801		
	MO 3	0.734		
Attention (AT)	AT 1	0.798	0.8791	0.5932
	AT 2	0.836		
	AT 3	0.726		
	AT 4	0.762		
	AT 5	0.723		
Open-mindedness (OM)	OM 1	0.804	0.839	0.6347
	OM 2	0.789		
	OM 3	0.797		

#### Discriminant validity analysis of the framework

The discriminant validity of the framework could be ensured by testing the correlation matrix among dimensions. Schumacker and Lomax (2016) proposed that in the structural discriminant validity analysis of tools, the AVE square root of all factors must be more than the absolute value of the Pearson correlation coefficient between two factors in order to be recognized as having discriminant validity. Therefore, as shown in Table 8, the result of structural discriminant validity analysis indicated that this framework had good discriminant validity.

**Table 8:** The results of interrelated coefficient matrix and square roots of AVE.

Construct	DC	OR	CL	RE	MO	AT	OM
DC	0.757						
OR	0.375***	0.732					
CL	0.565***	0.495***	0.778				
RE	0.421***	0.404***	0.531***	0.843			
MO	0.753***	0.545***	0.612***	0.455***	0.764		
AT	0.035	0.109*	0.138**	0.136**	0.113*	0.770	
OM	0.565***	0.545	0.585***	0.459***	0.758***	0.220***	0.797

***Significant at the 0.001 level; **Significant at the 0.01 level; *Significant at the 0.05 level.

### Item analysis

Item analysis was conducted to determine how well the items discriminate between college students with high abilities and those with low abilities in terms of CT within the scope of the item validity of the CT-evaluation scale form. In order to accomplish this goal, item discrimination statistics were calculated based on the differences between the lowest group means of 27% and the highest group means of 27% of the participants determined according to the scores of each item and to the total scores of the scale (Aridag and Yüksel, 2010). Therefore, first, the total scores for everyone were calculated by using the scale. This was followed by the calculation of total scores that were then ranked from the highest to the lowest. Of all the participants constituting the study group (*N* = 642), 27% (174) of them who had the highest scores were determined to be the higher group, and 27% of all the participants who had the lowest scores were determined to be the lower group. The independent samples *t*-test was applied for the purpose of statistically testing the difference between the mean scores of the two groups. The results obtained are presented in Table 9. Further, items with dimensional Pearson correlation coefficients and standardized factor loadings that did not reach the standard value (less than 0.4 and 0.45 respectively) were eliminated. Finally, for the remaining 22 items, the decisive values were higher than 0.3, and the gross interrelated coefficient between questions and items was higher than 0.4. Overall, the item analysis results showed that the remaining 22 items reached the standard.

**Table 9:** *t*-test results for the item means of the high-low-27% group.

Item number	Groups	*N*	Mean	*SD*	*df*	*t*	*p*
Item1	Lower Group	174	2.39	0.858	346	13.290	0.001
	Higher Group	174	3.57	0.807			
Item2	Lower Group	174	2.46	0.830	346	18.552	0.001
	Higher Group	174	4.02	0.733			
Item3	Lower Group	174	2.55	0.850	346	13.295	0.001
	Higher Group	174	3.81	0.915			
Item4	Lower Group	174	2.48	0.795	346	14.736	0.001
	Higher Group	174	3.85	0.938			
Item5	Lower Group	174	2.43	0.869	346	15.990	0.001
	Higher Group	174	3.97	0.934			
Item6	Lower Group	174	3.51	0.781	346	13.689	0.001
	Higher Group	174	4.50	0.546			
Item7	Lower Group	174	3.63	0.842	346	11.627	0.001
	Higher Group	174	4.54	0.605			
Item8	Lower Group	174	3.63	0.842	346	13.697	0.001
	Higher Group	174	4.66	0.532			
Item9	Lower Group	174	3.51	0.911	346	7.747	0.001
	Higher Group	174	4.21	0.787			
Item10	Lower Group	174	3.68	0.810	346	8.710	0.001
	Higher Group	174	4.46	0.851			
Item11	Lower Group	174	3.66	0.870	346	8.781	0.001
	Higher Group	174	4.45	0.801			
Item12	Lower Group	174	3.72	0.908	346	8.509	0.001
	Higher Group	174	4.50	0.788			
Item13	Lower Group	174	3.45	0.822	346	8.714	0.001
	Higher Group	174	4.17	0.725			
Item14	Lower Group	174	2.48	0.885	346	7.768	0.001
	Higher Group	174	3.37	1.227			
Item15	Lower Group	174	3.01	1.020	346	11.980	0.001
	Higher Group	174	4.27	0.938			
Item16	Lower Group	174	2.55	0.808	346	15.371	0.001
	Higher Group	174	3.94	0.878			
Item17	Lower Group	174	3.20	0.758	346	15.199	0.001
	Higher Group	174	4.28	0.552			
Item18	Lower Group	174	3.13	0.787	346	14.166	0.001
	Higher Group	174	4.24	0.669			
Item19	Lower Group	174	3.28	0.726	346	16.216	0.001
	Higher Group	174	4.43	0.582			
Item20	Lower Group	174	3.34	0.801	346	15.050	0.001
	Higher Group	174	4.47	0.586			
Item21	Lower Group	174	3.18	0.791	346	13.859	0.001
	Higher Group	174	4.28	0.675			
Item22	Lower Group	174	3.05	0.832	346	12.023	0.001
	Higher Group	174	4.09	0.782			
Total	Lower Group	174	56.61	4.13	346	42.881	0.001
	Higher Group	174	78.33	5.25			

***Significant at the 0.001 level; **Significant at the 0.01 level; *Significant at the 0.05 level.

## Discussion

CT is one of the key competencies that college students need to acquire (Bandyopadhyay and Szostek, 2019). This study aimed to construct a self-evaluation CT framework for college students in the humanities. In the initial framework, three dimensions and 27 items were conceived; then EFA was conducted, and items with independent factor loadings below 0.5 were excluded (Fabrigar et al., 1999). As a result, 25 items were retained for CFA. The results showed that three items should be eliminated because of their lower standard load (less than 0.5). Subsequently, the evaluation model with 22 items had an acceptable fitting index; meanwhile, good convergence and discriminant validity of the framework was also shown by calculating CR, AVE, and the square roots of AVE. Finally, to verify the suitability and distinctiveness of the constructed items, item analysis was conducted. The result showed that for the remaining 22 items, the decisive values were higher than 0.3, and the gross interrelated coefficient between questions and items was higher than 0.4, so the remaining 22 items reached the standard. Therefore, the final self-evaluation CT framework is a 22-item instrument, measuring three dimensions and six sub-dimensions: discipline cognition, CT disposition (open-mindedness, motivation, and attentiveness), and CT skills (reflection, organization skills, and clarification skills).

Compared to previous studies about the construction of an assessment framework for CT, this study focused on three important issues: the CT abilities of college students majoring in the humanities was the focus of this study; both CT skills and CT dispositions were included; and more specific dimensions of CT were the core measurement factors. In previous CT assessment frameworks, students in the disciplines of science (mathematics, business, nursing, engineering, etc.) were often the main subjects of study (Kim et al., 2014; Michaluk et al., 2016; Siew and Mapeala, 2016; Basri and Rahman, 2019), while college students majoring in the humanities have received less attention. However, CT as a guide of belief and action (Gyenes, 2021) is an important ability for college students in all fields (Davies, 2013; Zhang et al., 2022). In humanities subjects, research has shown that independent thinking skills are valuable indicators of students’ discipline-specific abilities in humanities subjects (Bertram et al., 2021). College students in the humanities need CT abilities to identify problems and find critical solutions (Baş et al., 2022). Meanwhile, the assessment instrument developed in this study added the dimension of disciplinary cognition, which is considered a prerequisite to help college students have a clear idea of their subject background. Therefore, the CT assessment framework provided a practical method for teachers and learners in the humanities to investigate the level of their CT abilities. For example, in the discipline of history, thematic history projects could be applied to foster students’ CT abilities in authentic history teaching contexts (Yang, 2007). In order to verify whether the projects help to improve learners’ CT abilities, this CT evaluation framework can be applied before and at the end of the project to determine whether there are differences in learners’ levels of CT abilities before and after learning. Likewise, in philosophy classroom, philosophical whole-class dialog can be useful teaching strategies to activate learners to think critically about moral values (Rombout et al., 2021). Learners in dialogs must take others’ perspectives into account (Kim and Wilkinson, 2019), which is in line with the sub-dimension of open-mindedness in the current CT evaluation framework. Hence, the CT evaluation framework can also be applied in specific disciplines.

In addition, in the current CT evaluation framework, both CT skills and CT dispositions were included, and more specific dimensions of CT were the core measurement factors. In terms of CT disposition, it reflects the strength of students’ belief to think and act critically. In the current evaluation instrument, the three sub-dimensions of motivation, open-mindedness, and attentiveness are the evaluation factors. The cultivation of college students’ CT abilities is usually based on specific educational activities. When college students get involved in learning activities, there are opportunities for them to foster their CT abilities (Liu, 2014; Huang et al., 2022). An important factor influencing student engagement is motivation (Singh et al., 2022), which has an important effect on college students’ behavior, emotion, and cognitive process (Gao et al., 2022). Hence, it makes sense to regard motivation as a measure factor of CT disposition, and it is crucial for college students to self-assess their motivation level in the first place to help them have a clear insight into their overall level of CT. The sub-dimension of attentiveness was also an important measurement factor, which aimed to investigate the level of the persistence of attention. Attentiveness also has a positive influence on a variety of student behaviors (Reynolds, 2008), while the sub-dimension of open-mindedness mainly assesses college students’ flexibility of thinking, which is also an important factor of CT (Southworth, 2020). A good critical thinker should be receptive of some views that might be challenging to their own prior beliefs with an open-minded attitude (Southworth, 2022). CT skills were then assessed in the following three sub-dimensions of clarification skills, organization skills, and reflection, with the aim of understanding how well students use CT skills in the problem-solving process (Tumkaya et al., 2009). The three sub-dimensions of CT skills selected in this framework are consistent with the specific learning process of problem solving, which begins with a clear description and understanding of the problem, i.e., clarification skills, followed by the ability to extract key information about the problem, to organize and process it, and to organize the information with the help of organizational tools such as diagrams and mind maps. Finally, the whole process of problem solving is reflected upon and evaluated, and research has shown that reflection learning intervention could significantly improve learners’ CT abilities (Chen et al., 2019).

In other words, the self-evaluation framework of college students’ CT constructed in this study focused on the investigation of college students in the humanities, and the descriptions of specific items combined the characteristics of the humanities. What’s more, because there are some differences in the extent to which students apply specific CT skills and are aware of how to use CT to solve problems based on their different disciplinary backgrounds (Belluigi and Cundill, 2017), the construction of the CT assessment framework for college students provides a practical pathway and a more comprehensive instrument for assessing the CT abilities of college students majoring in the humanities, and a research entry point was provided for researchers to better research the CT of college students majoring in the humanities.

## Conclusion

Based on a previous literature review of CT, this study further investigated the necessity of college students’ CT to construct a framework for evaluating the CT of college students in the humanities, and to test its effectiveness. The EFA, CFA, and item analysis methods were conducted in this study to construct a three-dimensional college students’ CT self-evaluation framework. The results indicate that the framework constructed in this study has good reliability and validity. Finally, a framework with three dimensions (discipline cognition, CT disposition, and CT skills) and seven sub-dimensions (discipline cognition, motivation, attentiveness, open-mindedness, reflection, organization skills, and clarification skills) totaling 22 items was developed.

### Implications

The main significance of this study is reflected in three aspects. Firstly, the current study constructed a CT-evaluation framework for college students majoring in the humanities. The results of the EFA, CFA, and item analysis supported the reliability and validity of the three-dimensional framework which indicates that it consists of discipline cognition, CT disposition, and CT skills. The specific assessment factors not only integrate the two dimensions of CT (skills and disposition), making the assessment framework more comprehensive, but also integrate the dimension of discipline cognition, enabling specific measures to be developed based on specific disciplinary contexts, ensuring that CT is assessed more accurately and relevantly. Second, the CT-evaluation framework can be applied in specific instruction and learning contexts. It is well known that CT has become one of the abilities in the 21st century. In instruction and learning, specific instructional strategies and learning activities should be purposefully applied according to specific humanistic backgrounds. Prior to undertaking specific teaching activities, it is worth having a prerequisite understanding of college students’ level of CT abilities by inviting students to complete the self-evaluation CT competence instrument. Likewise, after the learning activities, it is also an important instrument to evaluate the effectiveness of learning activities in terms of cultivating college students’ CT abilities. Finally, the construction of the CT assessment framework for college students provides a practical pathway for assessing the CT abilities of college students majoring in the humanities, and a research entry point was provided for researchers to better research the CT of these students majoring in the humanities in the future.

### Limitations and future work

There are two main limitations of this study. First, the sample in this study was from one area and was selected by random sampling, which cannot cover all the college students in the major. More and larger representative samples will be needed in the future to assess the extent to which the findings are applicable to other population groups to confirm the conclusions of the study. In addition, this evaluation framework of college students’ CT is still in the theoretical research stage and has not yet been put into practice. Therefore, the framework should be practically applied in further research to improve its applicability and usability according to practical feedback.

## Data availability statement

The original contributions presented in the study are included in the article/supplementary material, further inquiries can be directed to the corresponding author/s.

## Ethics statement

Ethical review and approval was not required for the study on human participants in accordance with the local legislation and institutional requirements. Written informed consent for participation was not required for this study in accordance with the national legislation and the institutional requirements.

## Author contributions

QL: conceptualization. SL: methodology. SL and ST: writing—original draft preparation. SL, XG, and QL: writing—review and editing. All authors have read and agreed to the published version of the manuscript.

## Funding

This study was supported by the School Curriculum Ideological and Political Construction Project (no. 1812200046KCSZ2211).

## Conflict of interest

The authors declare that the research was conducted in the absence of any commercial or financial relationships that could be construed as a potential conflict of inter.

## Publisher’s note

All claims expressed in this article are solely those of the authors and do not necessarily represent those of their affiliated organizations, or those of the publisher, the editors and the reviewers. Any product that may be evaluated in this article, or claim that may be made by its manufacturer, is not guaranteed or endorsed by the publisher.

## Footnotes

^1^ www.wjx.cn
